# In-Depth LC-ESI/HRMS-Guided Phytochemical Analysis and Antioxidant Activity Analysis of Eco-Sustainable Extracts of *Cynara cardunculus* (Carciofo di Paestum PGI) Leaves

**DOI:** 10.3390/plants13243591

**Published:** 2024-12-23

**Authors:** Antonietta Cerulli, Roberta Cuozzo, Maria Paola Melis, Gabriele Serreli, Monica Deiana, Milena Masullo, Sonia Piacente

**Affiliations:** 1 Dipartimento di Farmacia, Università degli Studi di Salerno, via Giovanni Paolo II n. 132, 84084 Fisciano, SA, Italy; rocuozzo@unisa.it (R.C.); mmasullo@unisa.it (M.M.); 2 Agritech National Research Center, Corso Umberto 40, 80138 Naples, CM, Italy; 3 PhD Program in Drug Discovery and Development, University of Salerno, Via Giovanni Paolo II 132, 84084 Fisciano, SA, Italy; 4 Dipartimento di Scienze Biomediche Unità di Patologia Sperimentale Cittadella Universitaria, Università degli Studi di Cagliari, S.P.8, 09042 Monserrato, CA, Italy; mpmelis@unica.it (M.P.M.); gabriele.serreli@unica.it (G.S.); mdeiana@unica.it (M.D.)

**Keywords:** “Carciofo di Paestum” PGI leaves, eco-sustainable extracts, antioxidant activity on Caco-2 cells, LC-ESI/HRMS analysis, NMR analysis, specialized metabolites

## Abstract

The Italian Carciofo di Paestum (*C. scolymus*) PGI, an artichoke variety from the Campania region, was investigated for its potential to reuse by-products for food supplements. EtOH:H_2_O 50:50 and 75:25 extracts of its leaves were analyzed for phenolic and flavonoid content and antioxidant activity (TEAC: 1.90 and 1.81 mM of Trolox; DPPH IC_50_: 106.31 µg/mL and 128.21 µg/mL; FRAP: 1.68 and 1.58 mM FeSO₄/g extract). To further investigate the antioxidant potential, the ability of the two extracts to scavenge reactive species was assessed in Caco-2 cell cultures, showing a dose-dependent antioxidant capacity. To highlight metabolites responsible for the activity, LC-ESI/HRMSMS analysis was achieved, revealing 28 compounds (sesquiterpenes, megastigmanes, quinic acid and hydroxycinnamic acid derivatives, flavonoids, lignans, triterpenoid saponins, and polar fatty acids), of which structures were determined using 1D- and 2D-NMR analysis. In addition, quantitative determination of caffeoyl, dicaffeoyl, and quinic acid derivatives (CQAs) was performed through LC-ESI/QTrap/MS/MS, highlighting that the most abundant compound was 5-caffeoylquinic acid (**6**), with values of 9.310 and 7.603 mg/g extract in EtOH:H_2_O (75:25) and EtOH:H_2_O (50:50), respectively. The analysis showed that extracts were rich in bioactive compounds, suggesting their potential for development into antioxidant-based food supplements that may protect cells from oxidative stress and support overall wellness.

## 1. Introduction

The globe artichoke, scientifically known as *Cynara cardunculus* var. *scolymus*, belongs to the Asteraceae family. It is a perennial plant highly valued for its edible immature flower buds, which are harvested before they fully bloom [1,2,3]. Flower buds are commonly referred to as “capitula” or “heads” and represent the edible part; they are consumed fresh, canned, or frozen [4,5,6].

Italy is a global leader in artichoke production, accounting for 390 Kt (26%) of the total worldwide production [7,8]. One of the most well-known cultivars of artichokes in Italy is “Carciofo di Paestum” PGI (Protected Geographical Indication), which customers and chefs prize for its unique flavor, delicacy, and size [9]. The main growing area for the above-mentioned cultivar is Paestum, a town in the Campania region, Southern Italy, known for its bountiful plains and rich agricultural heritage. The excellent taste and quality of this particular variety of artichoke are the result of the region’s special soil and environment [9].

External bracts and leaves, as well as stems, stalks, roots, and, to a lesser extent, seeds, represent by-products of industrial processing [10]. According to FAO estimates, 1.3 billion tons of edible food are lost or wasted annually, producing about 190,000 tons of by-products, with a significant financial impact on the global economy (USD 750 billion annually) [7]. The United Nations continues to promote recovery policies and circular economy strategies to reduce waste and encourage reuse, as the environmental and economic impacts of food waste disposal are growing concerns. Among artichoke by-products, residual leaves are rich in nutrients (carbohydrates and proteins) and bioactive compounds, which represent potential ingredients for foodstuffs, functional foods, and food supplements due to their functional and biological properties. The main specialized metabolites reported in artichoke leaves are flavonoids, sesquiterpenes, and caffeoyl, dicaffeoyl, and quinic acid derivatives (CQAs) [11,12]. These compounds are a subgroup of phenolic acids composed of quinic acid linked to one to four caffeoyl units. They are known for their hepatoprotective properties and potent antioxidant activity, which help scavenge free radicals and protect cells from oxidative damage [13,14,15].

Supported by these premises, for the first time, eco-sustainable extracts of “Carciofo di Paestum” PGI leaves were investigated in order to add value to artichoke leaf by-products as a source of bioactive phytochemicals. Food supplements are typically extracted using non-toxic solvents, such as ethanol and water. Ethanol, a widely used solvent, is biodegradable [16]. As a result, eco-friendly solvents, including water and aqueous ethanol solutions, are favored in extraction processes [17,18]; therefore, herein, two different eco-friendly mixtures were used.

In detail, two eco-sustainable EtOH:H_2_O (50:50 and 75:25) extracts of artichoke leaves have been investigated for the total phenolic and flavonoid content and the radical scavenging activity through 1,1-diphenyl-2-picrylhydrazyl (DPPH), Trolox Equivalent Antioxidant Capacity (TEAC), and ferric reducing/antioxidant power (FRAP) assays. Furthermore, a cell-based antioxidant in vitro test was also conducted on differentiated intestinal Caco-2 cells. In order to identify the compounds mainly responsible for the antioxidant activity, a phytochemical analysis was performed on both extracts. In the first step, eco-sustainable extracts were analyzed through liquid chromatography coupled to electrospray ionization and high-resolution mass spectrometry (LC–ESI/HRMS) in negative ion mode.

To assign the chemical structures of the compounds detected through the LC-ESI/HRMS analysis, a phytochemical analysis of the EtOH:H_2_O 50:50 extract was conducted, leading to the isolation and structural identification of the metabolites through 1D- and 2D-NMR experiments. Finally, considering the bioactivities reported for caffeoyl, dicaffeoyl, quinic acid derivatives (CQAs) [19], quantitative analysis through liquid chromatography coupled with tandem mass spectrometry using an ESI source and a hybrid triple quadrupole-linear ion trap mass analyzer (LC-ESI/QTrap/MS/MS) was performed.

## 2. Results and Discussion

### 2.1. Extraction of the “Carciofo di Paestum” PGI Leaves and Evaluation of Phenolic and Flavonoid Content and Antioxidant Activity by Spectrophotometric Assays

**Figure 1:**
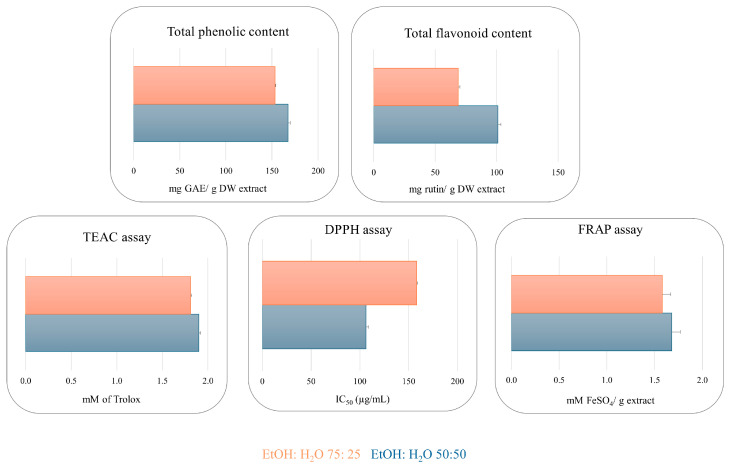
Total phenolic and flavonoid content, TEAC, DPPH, and FRAP assays of EtOH:H_2_O (50:50) (blue) and EtOH:H_2_O (75:25) (orange) extracts of “Carciofo di Paestum” PGI leaves.

The Folin–Ciocalteu assay was employed to determine the phenolic content. EtOH:H_2_O 50:50 and 75:25 extracts displayed a high concentration of phenolic compounds with a value of 167.48 and 153.41 (mg GAE/g extract), respectively. The same trend has been observed for the flavonoid content, with a value of 101.02 and 67.49 mg rutin/g extract value. A TEAC assay was performed successfully to obtain preliminary information about the radical scavenging properties of the extracts [20]. The extracts showed interesting antioxidant activity with a value of 1.90 and 1.81 (mM of Trolox) for EtOH:H_2_O 50:50 and EtOH:H_2_O 75:25, respectively, which is comparable to quercetin used as a reference compound (2.51 mM). The DPPH and FRAP assays displayed the same trend shown by the TEAC assay, with IC_50_ = 106.31 µg/mL and 128.21 µg/mL for DPPH and 1.68 and 1.58 mM FeSO_4_/g extract for FRAP assay (Figure 1 and Table S1). The remarkable antioxidant results, determined through spectrophotometric assays, confirm that flavonoids and phenolic derivatives contribute to the radical scavenging activity of the extracts (Figure 1 and Table S1) [21].

### 2.2. Cytotoxicity Evaluation in Caco-2 Cells Monolayers

**Figure 2:**
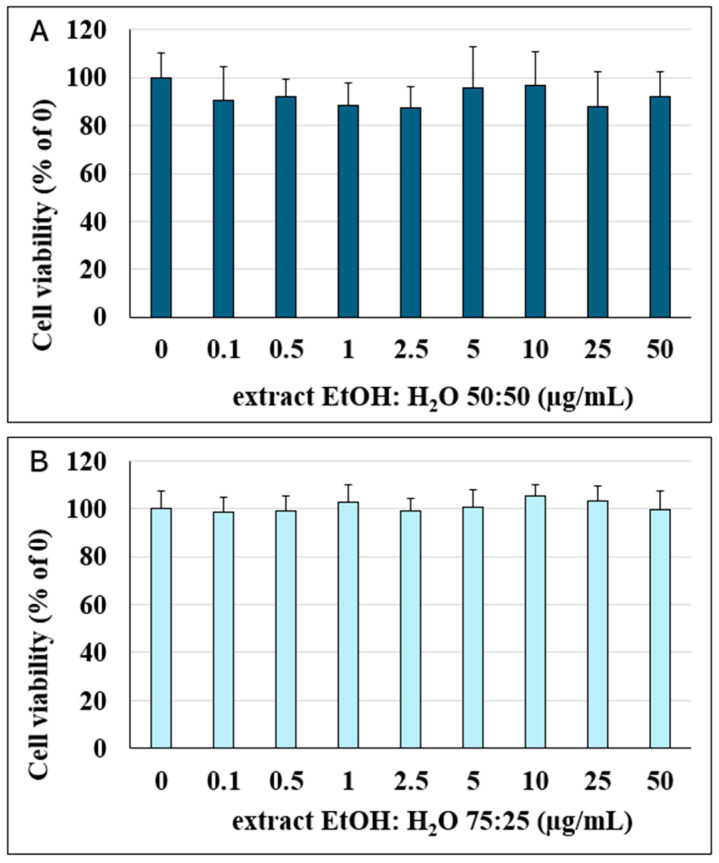
Percentage of viable Caco-2 cells calculated relative to the control (0 μM, 100% viability) following 24 h of incubation with different concentrations of EtOH:H_2_O (50:50) (**A**) and EtOH:H_2_O (75:25) (**B**) extracts (0.1–50 μM). Data are presented as the mean ± SD from independent experiments (n = 16).

Preliminarily, before evaluating the antioxidant capacity of the extracts on Caco-2 cells’ monolayers, cell viability was first assessed following treatment with different concentrations of the extracts. As shown in Figure 2, cell viability was not significantly affected by either extract at any of the concentrations tested (0.1–50 μM) after 24 h of incubation. Therefore, different concentrations of the extracts were tested to evaluate their antioxidant activity.

### 2.3. Antioxidant Activity Against TBH-Induced Oxidative Stress in Caco-2 Cells’ Monolayers

With the aim to confirm the antioxidant activity observed through spectrophotometric assays, an in vitro antioxidant test was conducted. Specifically, the capacity of the two extracts to scavenge reactive species was assessed in Caco-2 cell cultures by measuring intracellular ROS levels using 2′,7′-DCFH-DA.

**Figure 3:**
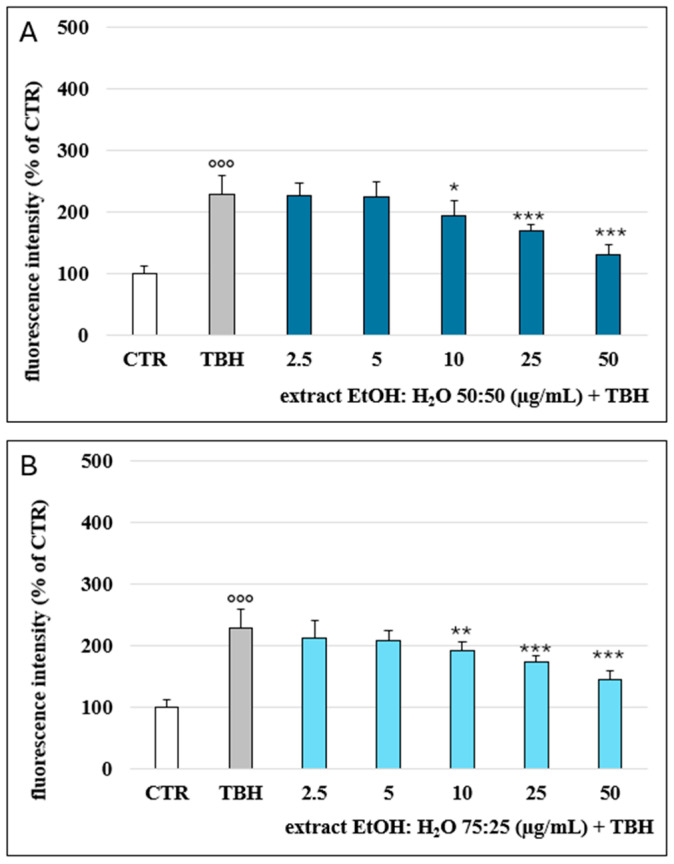
ROS levels, detected via H_2_-DCF-DA fluorescence and expressed as % of the control samples (CTR) in Caco-2 cells following 2 h of incubation with varying concentrations of the EtOH:H_2_O (50:50) (**A**) and EtOH:H_2_O (75:25) (**B**) (2.5–50 μM) in co-incubation with TBH 2.5 mM. °°° = *p* < 0.001 TBH vs. CTR; * = *p* < 0.05 extracts vs. TBH; ** = *p* < 0.01 extracts vs. TBH; *** = *p* < 0.001 extracts vs. TBH (n = 12).

Treatment with tert-butylhydroperoxide (TBH) (2.5 mM) resulted in a significant increase in ROS production (120% ROS increase at 2 h of incubation) in comparison with the non-treated cells (CTR), as reported in Figure 3. The pretreatment with the two extracts partially counteracted TBH-induced oxidative stress in a dose-dependent manner. The extracts, tested at 2.5 and 5 μg/mL, did not affect the oxidative stress but significantly inhibited ROS production at 10, 25, and 50 μg/mL. Substantially, no difference was noted between the two extracts tested, except for the EtOH:H_2_O (75:25) extract, tested at 10 μg/mL, which showed a higher significance (*p* < 0.01) than the EtOH:H_2_O (50:50) extract (*p* < 0.05). These data confirm the biological action of artichoke phenols as previously reported, where phenolic extracts of different parts of the artichoke were found to be active against oxidative stress and inflammation in the same cellular model [22,23,24].

### 2.4. LCESI/LTQOrbitrap/MS Analysis of “Carciofo di Paestum” Leaves’ Extracts

**Figure 4:**
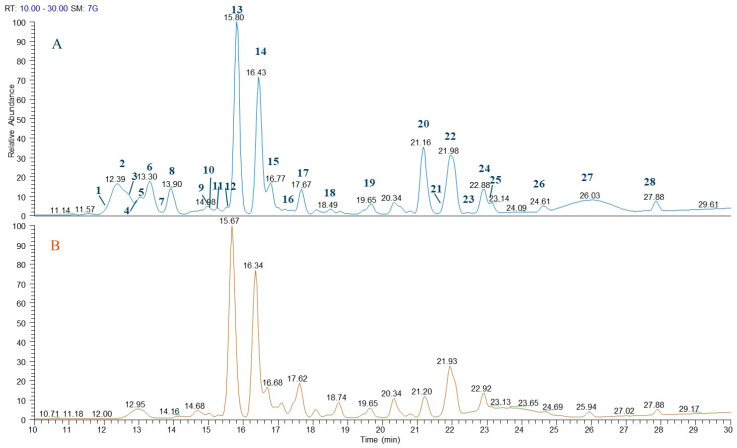
LC-ESI/HRMS profile of EtOH:H_2_O (50:50) (**A**) and EtOH:H_2_O (75:25) (**B**) extracts of “Carciofo di Paestum” PGI leaves.

In order to identify bioactive metabolites in the extracts of artichoke leaves, LC-ESI/HRMS was achieved. A detailed analysis of the LC-ESI/HRMS spectra led to the identification of 28 compounds belonging to the class of sesquiterpenes (**1**, **3**, **7**, **11**, **12**, **18**), megastigmanes (**4**, **15**), quinic acid derivatives (**2**, **6**, **8**, **9**, **10**, **17**), flavonoids (**13**, **14**, **20**), hydroxycinnamic acid derivatives (**5**, **19**, **23**), lignan (**16**), triterpenoid saponins (**21**, **25**), and polar fatty acids (**22**, **24**, **26**–**28**) (Figure 4 and Table 1).

Quinic acid derivatives and flavonoids represented the main classes of specialized metabolites. Compounds **2**, **6**, **8**, **9**, **10**, and **17** showed a specific fragmentation at *m*/*z* 191 (C_7_H_11_O_6_) due to [quinic acid-H]^−^; therefore, these compounds were assigned as quinic acid derivatives. They also displayed fragments indicating the nature of the moiety linked to quinic acid; in particular, compounds **2** and **6** showed a fragment at *m*/*z* 179 (C_9_H_7_O_4_) corresponding to [caffeoyl-H]^−^ and, consequently, they were assigned as caffeoyl–quinic acid derivatives; compounds **9** and **17** showed fragments at *m*/*z* 353 (C_16_H_17_O_9_) and *m*/*z* 179 (C_9_H_7_O_4_) related to [caffeoyl-quinic acid-H]^−^ and [caffeoyl-H]^−^, respectively, and thus they were identified as dicaffeoyl–quinic acids. Finally, compounds **8** and **10** showed a fragment at *m*/*z* 163 (C_9_H_7_O_3_) and *m*/*z* 193 (C_10_H_9_O_4_) attributed to [cumaroyl-H]^−^ and [feruloyl-H]^−^, respectively, linked to quinic acid [25]. Regarding flavonoid derivatives, the analysis of the fragmentation spectra of each compound allowed for the identification of the flavonoid class and the presence of one or more sugars linked to the aglycone. In detail, compounds **13** and **14**, based on their fragmentation spectra, were assigned as luteolin 7-*O*-rutinoside and luteolin 7-*O*-β-D-glucopyranoside; according to the literature, both compounds displayed the base peak at *m*/*z* 285 corresponding to a molecular formula of C_15_H_9_O_6_ ascribable to [luteolin-H]^−^. Consequently, the loss of 308 Da and 162 Da corresponded to the dehydrated form of rutinoside and glucopyranoside [26,27]. Moreover, compound **20** was assigned as luteolin. All the above-mentioned compounds were previously reported in *C. scolymus* leaves [5,28] (Table 1).

By careful analysis of LC-ESI/HRMS spectra, compounds **1**, **3**, **7**, **11**, **12**, and **18** were tentatively identified as sesquiterpene derivatives. Compounds **21** and **25** were assigned as cynarasaponin E and C, respectively, previously described in artichoke leaves extracts [28,29,30] (Table 1). Continuing the analysis of LC-ESI/HRMS profile, compounds **4** and **15** were tentatively assigned as megastigmane derivatives; in particular, after fragmentation, a base peak originated by neutral loss of dehydrated form of a hexose unit (162 Da) due to a deprotonated megastigmane unit was observed. Herein, megastigmane derivatives (**4**, **15**) are reported for the first time in *Cynara* genus (Table 1). The LC-ESI/HRMS spectra of peak **16**, after fragmentation, showed a product ion ascribable to the deprotonated phenolic compound generated by neutral loss of the dehydrated form of a hexose unit (neutral loss of 162 Da); in fact, peak **16** showed as main base peak *m*/*z* 357 (C_20_H_21_O_6_) corresponding to [pinoresionl-H]^−^. To the best of our knowledge, compound **16** is here reported for the first time in *Cynara* genus. Furthermore, the analysis of the exact mass and fragmentation spectra of compounds **5**, **19**, and **23** allowed us to assign them as hydroxycinnamic acid derivatives, in detail; they were identified as eugenol-*O*-rutinoside, ferulic acid, and caffeoyl ethyl ester, respectively. Among these compounds, **5** is reported for the first time in *Cynara* genus. Finally, peaks **22**, **24**, and **26**–**28** were designated as polar fatty acids belonging to oxylipin derivatives, as previously reported by Cerulli et al., 2021 [20] (Figure 4 and Table 1).

### 2.5. Isolation and Identification of Specialized Metabolites

In order to characterize unambiguously the main compounds in the extracts, EtOH:H_2_O (50:50) extract was submitted to Sephadex LH-20 and purified through HPLC-UV and HPLC-RI. In this way, compounds **1**, **2**, **6**, **8**, **9**, **11**–**14**, **17**–**20**, and **23** were isolated, and their structures were determined through the combination of monodimensional and bidimensional NMR experiments and also comparing the results with the literature [9,31,32].

**Figure 5:**
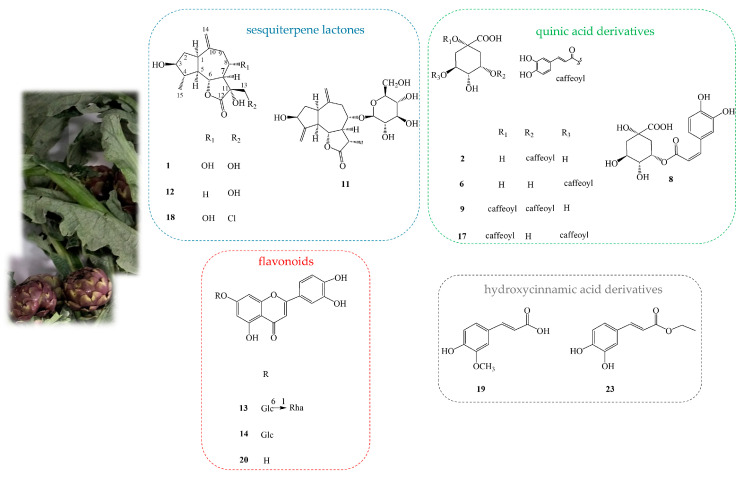
Specialized metabolites isolated from EtOH:H_2_O (50:50) extract of “Carciofo di Paestum” PGI leaves.

The ^1^H NMR spectrum of compound **1** showed the signals of three oxymethines at *δ* 3.64 (m), 4.08 (dd, *J* = 10.2, 4.2), and 4.16 (t, *J* = 10.3), an exomethylene at *δ* 5.12 (s), an oxymethylene at *δ* 3.77 (d, *J* = 10.2), and a signal at *δ* 1.23 (d, *J* = 6.0) ascribable to one methyl group (Table S2). In addition, the ^13^C NMR data of **1** displayed signals ascribable to a guaiane skeleton. The NMR data revealed the presence of 3β, 8α, 11α, 13- tetrahydroxy-10(14)-guaien-1α, 4β, 5α, 6βH-6α, and 12-olide (Table S2 and Figure 5). The ^1^H NMR spectra of **11**, **12**, and **18** (Table S2 and Figure 5) evidenced close similarities to compound **1**, so **11**, **12**, and **18** were assigned to the same class but with slight differences. In particular, the ^1^H NMR spectrum of compound **2** differed from **1** for the absence of the oximethine function at C-8 (Table S2). The NMR data of compound **8** pointed to a close similarity of those of **1**, with a likely replacement of the alcoholic group at C-13 of compound **1** with a chlorine atom in compound **18** (Table S2). The ^1^H NMR spectrum of compound **11** was superimposable to dodesacylcynaropicrin with additional signals ascribable to one hexose unit [30]; in particular, the careful analysis of 1D- and 2D-NMR spectra of **11** allowed us to assign a β-glucopyranosyl unit (*δ* 4.49) to C-8 (*δ* 84.8). In conclusion, compounds **11**, **12**, and **18** were assigned as dodesacyl-cynaropicrin 8-*O*-β-D-glucopyranoside, cynaratriol, and cynarinin B, respectively (Figure 5).

The careful analysis of the NMR spectra of compounds **2** and **6** allowed us to identify single caffeoyl-substituted quinic acid derivatives. The ^1^H NMR spectra of compounds **2** and **6** showed signals for one caffeoyl unit in each compound at *δ* 7.58 and 7.55 (d, *J* = 16.0 Hz), *δ* 6.30 and 6.28 (d, *J* = 16.0 Hz), *δ* 7.07 and 7.06 (d, *J* = 1.8 Hz), *δ* 6.96 and 6.98 (dd, *J* = 1.8, 8.0), and *δ* 6.76 and 6.82 (d, *J* = 8.0 Hz), respectively, and signals ascribable to a quinic acid unit [33] (Table S3). The position of the caffeoyl substitution was determined by analyzing the chemical shifts and coupling constants of the oxygenated methine protons in the quinic acid core. Typically, the H-3 signal shows a small coupling constant and appears as a brd or br-type peak. In contrast, the H-5 signal appears as a ddd with key coupling constants (approximately 8.0, 8.0 and 3.0 Hz) [33] (Table S3, Figure 5). Due to caffeoyl acylation, the proton signal is down-field shifted [33]. Based on these rules, compounds **3** and **6** were assigned to 3-caffeoyl quinic acid, and 5-caffeoyl quinic acid, respectively (Figure 5).

The ^1^H NMR spectra of compounds **9** and **17** displayed similar signals to compounds **2** and **6**, with an additional set of caffeoyl signals; therefore, compounds **9** and **17** were established as double caffeoyl-substituted quinic acid derivatives. Similarly to caffeoyl quinic acid derivatives, the acylation positions were determined by analyzing the chemical shifts and coupling constants of the oxygenated methine protons in the quinic acid core (Table S3 and Figure 5).

Compounds **13**, **14**, and **20** showed in 1D- and 2D-NMR spectra the typical chemical shifts of luteolin aglycone [9]; in addition, NMR experiments of compounds **13** and **14** exhibited, in the sugar region, signals of rutinoside and glucopyranoside moiety; consequently, the careful analysis of their NMR spectra allowed us to assign compounds **13**, **14**, and **20** as luteolin 7-*O*-rutinoside, luteolin 7-*O*-β-glucopyranoside, and the aglycone luteolin, respectively. Finally, the NMR analysis of compounds **19** and **23** permitted us to attribute these compounds to ferulic acid and caffeoyl-ethyl ester, respectively [31,32] (Figure 5).

### 2.6. Quantitative Analysis of Caffeoyl Quinic Derivatives (***2***, ***6***, ***9***, ***17***) in the Eco-Sustainable Extracts of Artichoke Leaves

With the aim of accurately quantifying CQAs in different eco-sustainable extracts, LC-ESI/QTrap/MS/MS analysis was performed. For this purpose, Multiple Reaction Monitoring (MRM), a highly precise tandem mass spectrometry technique, was used to assess the amount (mg/g of extract) of each compound in the extracts based on the selected transitions for the MRM experiments [34,35]. The MRM transitions were chosen on the basis of the fragmentation pattern shown by each metabolite in the ESI/MS/MS spectrum. Therefore, for the [M−H]^−^ pseudomolecular ion at *m*/*z* 353 (**2**, **6**), the transition 353→191 corresponding to [quinic acid-H]^−^ was chosen for MRM analysis [36,37]. In turn, the [M−H]^−^ pseudomolecular ion at *m*/*z* 515 (**9**, **17**) showed a main product ion at *m*/*z* 353 due to [caffeoyl quinic acid-H]^−^. Therefore, the transition 515→353 was chosen for MRM analysis of compounds **9** and **17** [38]. The most abundant compound in both extracts was 5-caffeoyl quinic acid (**6**), with values of 9.310 and 7.603 mg/g extract in EtOH:H_2_O (75:25) and EtOH:H_2_O (50:50), respectively, followed by 1,5-dicaffeoyl quinic acid (**17**), which occurred in EtOH:H_2_O (75:25) and EtOH:H_2_O (50:50) extracts at concentrations of 2.410 and 2.300 mg/g extract, respectively; compounds **2** and **6** were present in both extracts in very low concentrations (Table 2 and Table S4).

## 3. Materials and Methods

### 3.1. Sample Collection and Extraction

Leaves of *C. cardunculus* subsp. *scolymus*. cv. “Carciofo di Paestum” PGI were collected at Paestum, Salerno, Italy, in March 2023. Successively, leaves were dried on the bench at room temperature and in an oven at 30 °C. In the second step, to increase the surface of the leaf in contact with the solvent, the dried leaves were reduced into small pieces using a knife. The extracts were prepared using EtOH:H_2_O (75:25, 50:50) as solvents. In detail, 10 g of dried *C. scolymus* leaves were extracted with 200 mL of solvent mixture at room temperature (3 days, three times). After filtration, the solvent was evaporated with rotavapor to afford 2.20 g and 3.24 g of EtOH:H_2_O (75:25) and EtOH:H_2_O (50:50) of dried extracts, respectively.

### 3.2. Total Phenolic Content, Total Flavonoid Content, TEAC Assays, FRAP Assays

Folin–Ciocalteu, DPPH, TEAC, and FRAP assays for each extract, repeated in triplicate, were performed as previously reported, with slight modifications [9,39,40,41,42].

### 3.3. Cell Culture

The Caco-2 cell line was obtained from ECACC (Salisbury, UK) and treated as reported in the Supplementary Materials [43].

### 3.4. MTT Viability Test

The viability test was estimated using an MTT assay, as previously reported [43], to find any cytotoxic activity of the extract in differentiated Caco-2 cells (Salisbury, UK) (21 days post-seeding) (Supplementary Materials).

### 3.5. Determination of Intracellular Reactive Oxygen Species (ROS) Production

ROS release in Caco-2 cells was evaluated using the fluorescent probe H2-DCF-DA, as reported in a previous paper by Deiana et al. [44], with minor modifications (Supplementary Materials). For the extracts, a range of 2.5–50 μg/mL was tested.

### 3.6. Statistical Analyses

Data were analyzed by means of software GraphPad Prism 5 (GraphPad software, San Diego, CA, USA), using one-way analysis of variance (ANOVA) followed by post hoc Tukey’s test. Levels of *p* < 0.05 were considered statistically significant.

### 3.7. LC-ESI/HRMSMS Analysis of “Carciofo di Peastum” PGI Leaves Extracts

The extracts of *C. cardunculus* subsp. *scolymus* leaves were analyzed, in negative ion mode, through LC-ESI/HRMS using an HPLC with a hybrid mass spectrometer combining the linear trap quadrupole (LTQ) and the Orbitrap mass analyzer (ThermoScientific, San Jose, CA, USA); the same volume of 5 μL (concentration 1 mg/mL) and the same HPLC conditions were used for both samples (Supplementary Materials).

### 3.8. Isolation of Specialized Metabolites and ^1^H NMR Data Analysis

The EtOH:H_2_O (50:50) extract of the leaves was directly submitted to RP-HPLC-UV separation, allowing for the purification of compounds **2**, **6**, **9**, and **17**. Moreover, 3 g of the same extract was fractionated with Sephadex LH-20, and 80 fractions were obtained. Different fractions were purified through HPLC-RI, allowing for the purification of compounds **1**, **11**–**14**, and **18**. Other fractions were purified with RP-HPLC-UV in the same condition applied to the HPLC-UV of the extract, allowing for the purification of compounds **8**, **19**, and **23**. Fraction 78 corresponded to compound **20** (3.1 mg) (Supplementary Materials).

After purification, each compound was diluted in methanol-*d*_4_ (Merck, Milan, Italy) using a 5 mm tube, and NMR spectra were performed on a Bruker Ascend-600 spectrometer (Bruker BioSpin GmBH, Rheinstetten, Germany). Monodimensional and bidimensional NMR experiments were acquired (Supplementary Materials).

### 3.9. Quantitative Analysis of Caffeoyl, Dicaffeoyl, and Quinic Acid Derivatives (CQAs)

Quantitative analyses of CQAs derivatives were achieved, in triplicate, on an LC-ESI/QTrap/MS system (Sciex, Milan, Italy) using MRM mode. Specific chromatographic conditions were applied (Supplementary Materials). Different solutions of ES (0.001, 0.01, 0.1, 2.5, 10.0, 12.0, 15.0, and 17.0 µg/mL) were employed. The chromatographic method was assessed for calibration curve linearity, accuracy, and both intra- and inter-day precision. In detail, the acquisition was achieved three times in the same day (intraday) and in three different days (interday). The limit of quantification (LOD) and the limit of detection (LOD) were calculated [45].

## 4. Conclusions

In this study, the potential use of two eco-sustainable extracts (EtOH:H_2_O 50:50 and 75:25) of “Carciofo di Paestum” leaves for the development of food supplements was investigated.

EtOH:H_2_O 50:50 and 75:25 extracts showed high phenolic concentrations, with values of 167.48 mg GAE/g and 153.41 mg GAE/g, respectively. Flavonoid content followed a similar trend, with 101.02 mg rutin/g and 67.49 mg rutin/g. Moreover, the results showed the interesting antioxidant potential of the extracts across the different methods used for evaluation, with values of 1.90 mM and 1.81 mM for TEAC and an IC_50_ of 106.31 µg/mL and 128.21 µg/mL for DPPH and for EtOH:H_2_O 50:50 and 75:25, respectively. Finally, the FRAP assay showed a similar trend with values of 1.68 mM of FeSO_4_/g for EtOH:H_2_O 50:50 extract and 1.58 mM of FeSO_4_/g for EtOH:H_2_O 75:25 extract. To confirm the radical scavenging activity, an in vitro test on Caco-2 cell cultures was performed, revealing a dose-dependent antioxidant effect of both extracts, starting at 10 μg/mL. Qualitative analysis of both extracts through LC-ESI/HRMSMS was performed to identify the bioactive compounds potentially responsible for the antioxidant activity. In this way, 28 compounds were identified, mainly belonging to sesquiterpenes, quinic acid derivatives, and flavonoids, of which the structures were unambiguously assigned through 1D- and 2D-NMR. Among the identified compounds, our attention was focused on caffeoyl, dicaffeoyl, and quinic acid derivatives (CQAs), key markers of *C. scolymus* known for their wide-ranging therapeutic properties, including antioxidant, antibacterial, anticancer, hepatoprotective, cardioprotective, anti-inflammatory, antipyretic, neuro-protective, antiviral, antimicrobial, anti-hypertensive, and free radical scavenging effects [15,46,47]. Therefore, quantitative analysis of CQAs was performed using LC-ESI/QTrap/MS/MS. This analysis indicates that both eco-sustainable extracts contain a remarkable level of these bioactive compounds, supporting their potential for development into antioxidant-based food supplements.

## Figures and Tables

**Table 1:** Metabolites occurring in “Carciofo di Paestum” PGI leaves’ extracts based on LC-ESI/HRMS.

n°	Compound	*R*_t_ * (Min)	Molecular Formula	[M-H]^−^	[(M + HCOOH)-H]^−^	Δ ppm	Characteristic Product Ions
**1**	3β, 8α, 11α, 13-tetrahydroxy-10 (14)-guaien-1α, 4β, 5α, 6βH-6α, 12-olide	12.09	C_15_H_22_O_6_		343.1396	2.66	297.1339 (C_15_H_21_O_6_)
**2**	3-caffeoyl quinic acid	12.35	C_16_H_18_O_9_	353.0874		1.00	191.0559 (C_7_H_11_O_6_), 179.0348 (C_9_H_7_O_4_), 173.0348 (C_7_H_9_O_5_), 135.0452 (C_8_H_7_O_2_)
**3**	Cynarascoloside A/B	12.91	C_21_H_32_O_9_		473.2024	0.68	427.2024 (C_21_H_31_O_9_), 179.0558 (C_6_H_11_O_6_)
**4**	Megastigmane-*O*-hexoside	13.00	C_19_H_32_O_8_		433.2074	1.40	387.2016 (C_18_H_29_O_6_); 225.1003 (C_13_H_21_O_3_)
**5**	Eugenol-*O*-rutinoside	13.12	C_22_H_32_O_11_	471.1869		1.64	163.1105 (C_10_H_11_O_2_)
**6**	5-caffeoyl quinic acid (chlorogenic acid)	13.30	C_16_H_18_O_9_	353.0876		2.38	191.0562 (C_7_H_11_O_6_), 179.0313 (C_9_H_7_O_4_),
**7**	Cynarascoloside A/B	13.80	C_21_H_32_O_9_		473.2025	1.56	427.2025 (C_21_H_31_O_9_), 179.0558 (C_6_H_11_O_6_)
**8**	5-*p*-coumaroyl quinic acid	13.90	C_16_H_18_O_8_	337.0922		1.17	191.0561 (C_7_H_11_O_6_), 163.0452 (C_9_H_7_O_3_)
**9**	1, 3-dicaffeoyl quinic acid	14.94	C_25_H_24_O_12_	515.1190		1.08	353.0873 (C_16_H_17_O_9_), 191.0567 (C_7_H_11_O_6_), 179.0375 (C_9_H_7_O_4_)
**10**	Feruloyl quinic acid	15.00	C_17_H_20_O_9_	367.1030		1.75	193.0602 (C_10_H_9_O_4_), 191.0561 (C_7_H_11_O_6_)
**11**	dodesacylcynaropicrin 8-*O*-β-D-glucopyranoside	15.07	C_21_H_30_O_9_		471.1865	0.92	425.1865 (C_21_H_29_O_9_), 179.0556 (C_6_H_11_O_6_)
**12**	cynaratriol	15.50	C_15_H_22_O_5_		327.1445	2.05	263.1300 (C_15_H_19_O_4_)
**13**	Luteolin 7-*O*-rutinoside	15.80	C_27_H_30_O_15_	593.1502		0.19	447.0923 (C_21_H_19_O_11_), 285.0400 (C_15_H_9_O_6_)
**14**	Luteolin 7-*O*-β-D-glucopyranoside	16.43	C_21_H_20_O_11_	447.0930		1.79	285.0401 (C_15_H_9_O_6_)
**15**	Megastigmandienone-*O*-hexoside	16.77	C_19_H_30_O_7_		415.1966	0.72	369.1916 (C_18_H_27_O_5_) 207.0586 (C_13_H_19_O_2_)
**16**	Pinoresinol-*O*-hexoside	16.77	C_26_H_32_O_11_	519.1864		0.60	357.1339 (C_20_H_21_O_6_)
**17**	1, 5-dicaffeoyl quinic acid	17.63	C_25_H_24_O_12_	515.1185		0.31	353.0873 (C_16_H_17_O_9_), 191.0558 (C_7_H_11_O_6_), 179.0455 (C_9_H_7_O_4_)
**18**	Cynarinin B	18.49	C_15_H_21_O_5_Cl		361.1052	1.05	315.0567 C_15_H_20_O_5_Cl
**19**	Ferulic acid	19.65	C_10_H_10_O_4_	193.0507		6.24	161.0242 (C_9_H_5_O_3_)
**20**	Luteolin	21.16	C_15_H_10_O_6_	285.0318		1.39	241.0503 (C_14_H_9_O_4_), 175.0398 (C_10_H_7_O_3_), 151.0037 (C_7_H_3_O_4_)
**21**	Cynarasaponin E	21.29	C_42_H_66_O_15_	809.4319		0.10	647.3795 (C_36_H_55_O_10_), 585.3792 (C_35_H_53_O_7_), 471.3471 (C_30_H_47_O_4_)
**22**	Trihydroxy octadecadienoic acid	21.93	C_18_H_32_O_5_	327.2171		1.53	229.1442 (C_12_H_21_O_4_)
**23**	Caffeoyl-ethyl ester	22.06	C_11_H_12_O_4_	207.0663		3.91	179.0305 (C_9_H_7_O_4_)
**24**	Trihydroxy octadecenoic acid	22.88	C_18_H_34_O_5_	329.2327		1.46	229.1441 (C_12_H_21_O_4_), 211.1338 (C_12_H_19_O_3_), 171.1022 (C_9_H_15_O_3_)
**25**	Cynarasaponin C	23.14	C_42_H_66_O_14_		839.4426	0.32	793.4374 (C_42_H_65_O_14_) 631.3845 (C_36_H_55_O_9_)
**26**	Dihydroxy-octadecatrienoic acid	24.60	C_18_H_30_O_4_	309.2069		3.20	291.1960 (C_18_H_27_O_3_), 239.1646 (C_14_H_23_O_3_), 171.1022 (C_9_H_15_O_3_)
**27**	Hydroxy-oxooctadecatrienoic acid	26.29	C_18_H_28_O_4_	307.1911		2.42	289.1799 (C_18_H_25_O_3_), 235.1331 (C_14_H_19_O_3_)
**28**	Hydroxy-octadecatrienoic acid	27.88	C_17_H_26_O_4_	293.1754		2.43	236.1049 (C_13_H_16_O_4_)

*R*_t_ *: Retention time of the LC-ESI/HRMS profile of EtOH:H_2_O (50:50) extract.

**Table 2:** Quantitative results of compounds **2**, **6**, **9**, and **17** (mg/g extract ± SD) in the eco-sustainable extracts of *Cynara cardunculus* (Carciofo di Paestum PGI) leaves.

Maceration	3-Caffeoyl Quinic Acid (2)	5-Caffeoyl Quinic Acid (6)	1,3-Dicaffeoyl Quinic Acid (9)	1,5-Dicaffeoyl Quinic Acid (17)
EtOH:H_2_O (50:50)	0.069 ± 0.003	7.603 ± 0.112	0.006 ± 0.001	2.300 ± 0.101
EtOH:H_2_O (75:25)	0.079 ± 0.007	9.310 ± 0.825	0.014 ± 0.003	2.410 ± 0.159

## Data Availability

The data presented in this study are available in the Supplementary Materials.

## Supplementary Materials

The following supporting information can be downloaded at https://www.mdpi.com/article/10.3390/plants13243591/s1. Plant material; isolation of specialized metabolites, LC-ESI/HRMS/MS analysis; NMR and data processing; total phenolic content, total flavonoid content, DPPH, TEAC, and FRAP assays; Table S1: Phenolic content and antioxidant activity of eco-sustainable extracts of “Carciofo di Paestum” leaves; quantitative analysis of caffeoyl, dicaffeoyl, and quinic acid derivatives (CQAs); Table S2. ^1^H (600 MHz) and ^13^C (150 MHz) NMR data of compounds **1**, **11**, **12**, and **18** (CD_3_OD); Table S3. ^1^H (600 MHz) and ^13^C (150 MHz) NMR data of compounds **2**, **6**, **9**, and **17** (CD_3_OD); Table S4. LC–MS/MS conditions for quantitation of caffeoyl, dicaffeoyl, and quinic acid derivatives (CQAs) in negative ion MRM mode.
