# Supplementary material for: In-Depth LC-ESI/HRMS-Guided Phytochemical Analysis and Antioxidant Activity Analysis of Eco-Sustainable Extracts of Cynara cardunculus (Carciofo di Paestum PGI) Leaves
Source: Plants (Basel). 2024 Dec 23;13(24):3591. doi: 10.3390/plants13243591 (PMC11679891; doi:10.3390/plants13243591)
Supplement: Supplementary file 1 [file plants-13-03591-s001.zip › plants-3354156-supplementary.pdf]

Supplementary materials

In-Depth LC-ESI/HRMS-Guided Phytochemical Analysis and Antioxidant Activity Analysis of Eco-Sustainable Extracts of *Cynara cardunculus* (Carciofo di Paestum PGI) Leaves

Antonietta Cerulli [1,2,*], Roberta Cuozzo [1,3], Maria Paola Melis [4], Gabriele Serreli [4], Monica Deiana [4], Milena Masullo [1] and Sonia Piacente [1,2,*]

[1] Dipartimento di Farmacia, Università degli Studi di Salerno, via Giovanni Paolo II n. 132, 84084 Fisciano, Italy; rocuozzo@unisa.it (R.C.); mmasullo@unisa.it (M.M.)

[2] Agritech National Research Center, Corso Umberto 40, 80138 Naples, Italy

[3] PhD Program in Drug Discovery and Development, University of Salerno, Via Giovanni Paolo II 132, 84084 Fisciano, SA, Italy

[4] Dipartimento di Scienze Biomediche Unità di Patologia Sperimentale Cittadella Universitaria, Università degli Studi di Cagliari, S.P.8, 09042 Monserrato, CA, Italy; mpmelis@unica.it (M.P.M.); gabriele.serreli@unica.it (G.S.); mdeiana@unica.it (M.D.)

* Correspondence: acerulli@unisa.it (A.C.); piacente@unisa.it (S.P.); Tel.: +39-089-969763 (S.P.)

Plant Material

Leaves of *C. cardunculus* subsp. *scolymus*. cv. "Carciofo di Paestum" were collected at Paestum, Salerno, Italy, in March 2023, and identified by Prof. V. De Feo (Department of Pharmacy, University of Salerno, Italy). A voucher specimen has been deposited in this Department.

Total Phenolic Content, Total Flavonoid Content, DPPH, TEAC and FRAP assays

For extracts, Total Phenolic Content, Total Flavonoid Content, DPPH and TEAC assay were carried out. The total phenolic content of the extracts was determined by Folin-Ciocalteu assay; gallic acid was used as the reference compound (calibration equation: y= 0.00204x- 0.0077, R^2 = 0.995). All the experiments were performed in triplicate, and results were expressed as the mean of gallic acid equivalents (GAEmg/g dried extract).

For the total flavonoid content of the extracts, rutin was used as a reference compound (calibration equation: y = 0.0012x + 0.1011 R^2 = 0.9979).

For the DPPH assay, the percentage of DPPH$^\bullet$ radical scavenging activity (%) was plotted against the extract concentration (μg/mL) to determine the IC_{50}. In brief, stock solutions (1 mg/mL) of all the obtained extracts were used in the range of 50–200 μg/mL, and an aliquot (37.5 μL) of the methanol solution containing different amounts of each extract was added to 1500 μL of daily prepared DPPH$^\bullet$ solution. Absorbance at 517 nm was measured immediately on a UV-visible spectrophotometer (Multiskans sky-high, Thermo Fisher Scientific, Milan, Italy). All the experiments were performed in triplicate. Range = 50–200 μg/mL was tested for each extract; vitamin C (concentration 1-25 ug/ml) was used as a positive control and analyzed by linear regression.

Radical scavenging activity was determined by TEAC assay. In particular, in the TEAC assay, the antioxidant activities of analyzed extracts (range= 1.80-2.03 mg/mL) were expressed as TEAC values in comparison with the TEAC activity of quercetin; TEAC values are expressed as concentration (mM) of a standard Trolox solution exerting the same antioxidant activity of a 1 mg/mL solution of the tested extract. All the experiments were performed in triplicate. For each well 15 μL of extracts at the concentration of 750, 500 and 200 μg/mL were added to 1500 mL of work solution (prepared with PBS, ABTS$^\bullet$ and $K_2S_2O_8$) and the absorbance at 734 nm was measured immediately on a UV-visible spectrophotometer (Multiskans skyhigh, Thermo Fisher Scientific, Milan, Italy).

Ferric-reducing antioxidant power was determined by FRAP assay. The FRAP assay was carried out according to the method described in the literature. Briefly, a solution consisting in a 10:1:1 ratio of 23 mM acetate buffer (pH 3.6), 10 mM of tripyridyl triazine (TPTZ) in HCl (40 mM), and FeCl3 (20 mM) respectively was prepared as FRAP reagent. The assay was performed in 96 multiwell plate. In each well 264 μL of FRAP reagent and 8 μL of extracts at different concentrations were placed. The reaction mixture was incubated at 37 °C for 30 min in dark conditions. Absorbance was read at 593 nm using a UV-visible spectrophotometer (Multiskans skyhigh, Thermo Fisher Scientific, Milan, Italy). The absorbance of the blank (FRAP reagent) was subtracted from all absorbances with the sample to determine the FRAP value for each sample. Trolox was used as reference compound.

Table S1. Phenolic and flavonoid content; DPPH and TEAC assays of eco-sustainable extracts of "carciofo di Paestum" leaves.

extract	Total Phenol Content[a] (mg GAE/g extract ± SD[b])	Total Flavonoid Content[c] (mg rutin/g extract ± SD[b])	TEAC[c] (mM of Trolox ± SD[b])	DPPH EC_{50}[d] (μg/mL ± SD[b])	FRAP (mmol Fe (II)/g extract)
**MAC50**	167.48 ± 2.56***	101.02± 2.47***	1.90 ± 0.02***	106.31 ± 1.59***	1.68 ± 0.11***
**MAC75**	153.41 ± 0.58***	67.49 ± 6.66***	1.81 ± 0.01***	128.21 ± 0.88***	1.58 ± 0.09***
**quercetin[d]**			2.51± 0.01***		
**Vitamin C**				14.55± 0.09***	
**Trolox**					4.87 ± 0.20***

[a] *Values are expressed as milligrams of gallic acid equivalents (GAE) per gram of dried extract (mg GAE/g dried extract);* [b] *SD: Results are expressed as a mean of three experiments; SD, standard deviation.;* [c] *Values are expressed as concentration (mM) of a standard Trolox solution exerting the same antioxidant activity of a 1 mg/mL solution of the tested extract, the concentration of extracts 0.25-1.0 mg/ml.* [d] *standard compound for TEAC assay.* [e] *Values are expressed as micrograms per milliliter (μg/mL), with concentrations of extracts 50-200ug/ml.* [f] *standard compound for DPPH assay.* ****p< 0.001, vs. control a one-way ANOVA followed by Dunnett's multiple comparison test*

Cell Culture

Caco-2 cells are derived from human colorectal adenocarcinoma which, once reaching confluence, spontaneously differentiate into normal enterocytes. Dulbecco's modified Eagle's medium (DMEM) with low glucose and with L-Arginin, phosphate-buffered saline (PBS) without $MgCl_2$ and $CaCl_2$, fetal bovine serum (FBS) and penicillin/streptomycin 1X were obtained from Euroclone (Milano, Italy). Caco-2 cells were grown in T75 flasks until their confluence reached 80%, at 37 ◦C in a 5% CO_2 humidified atmosphere in DMEM supplemented with 1% antibiotic/antimycotic solution (100 U/mL penicillin, 100 mg/mL streptomycin), and 10% FBS [21]. At passage 45–60, cells were removed from flasks by adding a trypsin solution at 1% and incubating at 37 ◦C for 5–10 min; Caco-2 cells were then collected, centrifuged and counted in a Bürker chamber and then seeded into 96-well plates at a concentration of 5 × 104 cells/mL for subsequent experiments. Cells were cultured replacing the medium twice weekly.

MTT viability test

The cells were seeded in 96-well plates (2.5 × 104 cells/mL, 100 µL in each well), incubated with different concentrations (0.1 – 50 µg/mL) of dried extracts dissolved in ethanol, or with an equivalent amount of ethanol (2.5%) for the controls, and were incubated for 24 h. At 24 h before treatment, the 10% serum supplemented medium was discarded and replaced with a medium with 2.5% serum. After incubation, the medium was removed and 100 µL of the MTT solution (5 mg/mL of MTT in PBS in fresh serum free medium, 8% and 92% respectively) was added and left for 6 h at 37 ◦C. The MTT/medium solution was then removed and 100 µL of DMSO was added to each well. Afterwards, the absorbance of each well was measured at 570 nm using a microplate reader (Infinite F200, Tecan, Salzburg, Austria). Cell viability was expressed as percentage of control (0 µg/mL) values.

LC-ESI/HRMS/MS Analysis

The extracts of *C. cardunculus* subsp.*scolymus* leaves were analyzed, in negative ion mode, by LC-ESI/LTQOrbitrap/MS. LC-HRMS analysis was performed on Luna 5u C18(2) 100 A (150x2 mm) column (Phenomenex, Aschaffenburg, Germany), using a flow rate of 0.2 mL/min. A binary solvent system was used (phase A: water with 0.1% formic acid, phase B: acetonitrile with 0.1% formic acid). The HPLC gradient started at 5% B, after 10 min, % B was at 20%, after 20 min it was at 60%, in 2 min % B arrived at 95%, holding it at this percentage for 5 min, before returning to the starting percentage. The autosampler was set to inject 4 µL of each extract (1.0 mg/mL MeOH). The auxiliary gas was set at 10 (arbitrary units), and the sheath gas was set at 50 (arbitrary units).

Isolation of specialized metabolites

EtOH:H_2O (50:50) extract obtained by maceration extraction was purified directly by RP-HPLC-UV setting the wavelength at 254 nm. The elution gradient was obtained using water with 0.1% formic acid as eluent A and acetonitrile with 0.1% formic acid as B; the run was carried out with a flow rate of 2.0 mL/ min. A Phenomenex Sinergy 10u Hydro-RP 80A

(250 mm × 10 mm, 10 micron) was used. The following HPLC gradient conditions were used: 0 min 5 % B, 25 min 100 % B, 30 min 5 % B, 40 min 5 % B, allowing the isolation of compounds **2** (1 mg, t_R = 9.59 min), **6** (1.3 mg, t_R = 12.67 min); **9** (1 mg, t_R = 13.77 min); and **17** (2.4 mg; t_R = 15.94 min). 3 g of the same extract were fractionated with Sephadex LH-20 and 80 fractions were obtained. Fractions 19-21 were purified by HPLC-RI using SymmetryPrep C18 7 µm Column (7.8x300 mm) using MeOH−H_2O (4:6) as mobile phase (flow rate 2.0 mL/min) allowing the purification of compounds **1** (1.5 mg, t_R =6.2 min) and **11** (1.3 mg, t_R =10.4 min). Fractions 25-26 were purified in the same condition to yield compounds **12** (1.2 mg, t_R =13. min) and **18** (1.6 mg, t_R =12.2 min). Fractions 31-33 were purified with RP-HPLC-UV, with the same condition applied for the HPLC-UV of extract, allowing the purification of compounds **8** (0.7 mg, t_R =12.34 min), **19** (1.1 mg, t_R =15.11 min), and **23** (2 mg, t_R =20.59 min). Fractions 42-43 and 52-53 were purified by HPLC-RI using SymmetryPrep C18 7 µm Column (7.8x300 mm) using MeOH−H_2O (4:6) as mobile phase (flow rate 2.0 mL/min) to yield compound **14** (2.6 mg, t_R = 14.5 min), and compound **13** (0.5 mg t_R = 15.0 min), respectively. Fraction 78 corresponds to compound **20** (3.1 mg).

NMR and Data Processing

NMR analyses were carried out on a Bruker Ascend-600 spectrometer (Bruker BioSpin GmBH, Rheinstetten, Germany) equipped with a Bruker 5 mm tube. For the isolated compounds, DQF-COSY, HSQC, and HMBC spectra were acquired in methanol-d_4 (99.95%, Sigma-Aldrich, Milan, Italy), and standard pulse sequences and phase cycling were used. The 1D and 2D NMR data were pro-cessed by TOPSPIN 3.2 software.

Table S2. ^{1}H (600 MHz) and ^{13}C (150 MHz) NMR data of compounds **1**, **11**, **12**, and **18** (CD_3OD).

	**1**		**11**		**12**		**18**	
Position	δ_C	δ_H (*J* in Hz)	δ_C	δ_H (*J* in Hz)	δ_C	δ_H (*J* in Hz)	δ_C	δ_H (*J* in Hz)
1	43.4	2.84 m	43.91	2.94 m	42.9	2.83 m	43.0	2.86 m
2	38.8	1.70, 2.05 m	38.8	1.68, 2.22 m	39.0	1.71, 2.8 m	38.6	1.72, 2.08 m
3	77.8	3.64 m	73.1	4.51 m	78.7	3.66 m	78.0	3.65 m
4	47.5	1.79 m	148.0		47.8	1.80 m	47.8	1.79 m
5	52.1	1.97 m	50.7	2.82 m	52.6	1.89 m	52.8	1.99 m
6	80.5	4.16 t (10.3)	81.0	4.14 t (10.3)	80.9	4.21 t (10.2)	82.1	4.18 t (10.3)
7	60.5	2.30 t (10.3)	55.3	2.24 m	55.8	1.89 m	61.3	2.41 m
8	71.5	4.08 dd (10.3, 4.2)	84.8	3.80 m	27.9	1.68, 2.06 m	70.8	4.01 dt (10.6, 4.3)
9	47.8	2.1, 2.8 m	43.2	2.44, 2.88 m	37.5	1.90, 2.68 m	47.6	2.11, 2.76 m
10	145.6		145.7		148.0		143.0	
11	79.6		41.8	2.76 m	77.02		78.9	
12	180.5		179.0		178.6		178.8	
13	60.8	3.77 d (10.2), 4.01 d (10.2)	16.3	1.42 d (7.2)	64.0	3.67 d (10.2), 4.02 d (10.2)	43.0	3.74 d (10.2), 4.12 d (10.2)
14	113.3	5.12 s	115.0	5.08 s	112.2	4.94 s	115.1	5.05 s
15	18.4	1.23 d (6.0)	111.3	5.30 s	18.2	1.25 d (6.6)	18.3	1.23 t (5.5)
			β-Glc (at C-8)					
1'			104.8	4.49 d (8.0)				
2'			74.5	3.49 dd (8.0				
3'			77.0	3.43 dd (9.0, 9.0)				
4'			70.6	3.44 dd (9.0, 9.0)				
5'			77.5	3.33 m				
6'			62.0	3.70, 3.91 m				

Table S3. ^{1}H (600 MHz) and ^{13}C (150 MHz) NMR data of compounds **2**, **6**, **9**, and **17** (CD_3OD).

	**2**		**6**		**9**		**17**	
Position	δ_C	δ_H (*J* in Hz)	δ_C	δ_H (*J* in Hz)	δ_C	δ_H (*J* in Hz)	δ_C	δ_H (*J* in Hz)
1	74.0		75.0		81.1		80.9	
2	36.9	1.96, 2.22 m	36.3	2.02, 2.22 m	32.9	2.87, 2.20 m	35.6	1.70, 2.10 m
3	69.1	5.36 d (3.0)	70.0	4.18 d (2.1)	73.0	5.36 d (2.1)	71.9	4.19 d (2.1)
4	72.1	3.65 dd (8.1, 2.6)	72.1	3.78 dd (8.1, 2.6)	75.3	3.61 dd (8.5, 2.6)	72.82	3.96 dd (8.5, 2.6)
5	70.1	4.13 ddd, (3.6, 8.1, 8.5)	70.5	5.36 ddd (3.6, 8.1, 8.5)	67.8	4.22 ddd (3.6, 8.5, 8.7)	71.6	5.36 ddd (3.6, 8.5, 8.7)
6	38.4	1.96, 2.26 m	37.	2.04, 2.22 m	41.3	1.83, 2.87 m	39.5	2.30, 2.80 m
7	176.0		175.7		176.6		174.5	
	caffeoyl (at C-3)		caffeoyl (at C-5)		caffeoyl (at C-3)		caffeoyl (at C-5)	
1'	126.4		126.4		127.4		127.5	
2'	113.5	7.07 d (1.8)	113.3	7.06 d (1.8)	115.4	6.98 d (1.8)	115.5	7.05 d (1.8)
3'	145.6		145.3		146.5		147.2	
4'	148.9		148.6		149.3		149.9	
5'	115.5	6.76 d (8.0)	115.3	6.82 d (8.0)	116.1	6.63 d (8.0)	116.2	6.74 d (8.0)
6'	121.5	6.96 dd (1.8, 8.0)	121.4	6.98 d (1.8, 8.0)	122.0	6.88 dd (1.8, 8.0)	123.62	6.89 dd (1.8, 8.0)
7'	145.6	7.58 dd (16.0)	145.3	7.55 dd (16.0)	147.2	7.51 d (16.0)	148.9	7.55 d (16.0)
8'	113.2	6.30 d (16.0)	113.3	6.28 d (16.0)	115.1	6.21 d (16.0)	115.2	6.28 d (16.0)
9'	168.89		168.99		167.8		168.7	
					caffeoyl (at C-1)		caffeoyl (at C-1)	
1''					127.4		127.65	
2''					115.5	6.81 d (1.8)	115.9	7.12 d (1.8)
3''					146.7		147.2	
4''					149.7		149.9	
5''					116.6	6.50 d (8.0)	117.3	6.72 d (8.0)
6''					123.0	6.74 dd (1.8, 8.0)	123.7	6.86 dd (1.8, 8.0)
7''					147.8	7.46 d (16.0)	149.0	7.58 d (16.0)
8''					115.1	6.11 d (16.0)	115.3	6.18 d (16.0)
9''					167.9		168.0	

Quantitative analysis of caffeoyl-, dicaffeoyl quinic acid derivatives (CQAs)

Quantitative analyses was performed on an LC-ESI/QTrap/MS system working in MRM mode. HPLC separation was conducted by Kinetex Omega μm RP C18 column (100 mm× 2.1 mm i.d) at a flow rate of 0.3 μL/min.

Linear gradient elution was carried out by using H_2O with 0.1% formic acid as eluent A and acetonitrile with 0.1% formic acid as B. The HPLC gradient started at 5% B after 2.1 min % B was at 15%, changing from 15% B to 35% B in 4.3 min, from 35% B to 80% B in 2.10 min, returning to the starting percentage in 2.1 min. The instrument operated in the negative ion mode, resveratrol was used as internal standard (, 1μg/mL for each solution). *C. cardunculus* subsp. *scolymus* extracts were diluted by using methanol and 5 μL (1.0 mg/mL) were injected in triplicate; solutions of different ES concentrations (0.001, 0.01, 0.1, 2.5, 10.0, 12.0, 15.0, and 17.0 μg/mL) were used. Linear regression analysis was performed using the Analyst 1.6.2 Software provided by the manufacturer (AB Sciex, Milan, Italy).

Linearity was evaluated by correlation values of calibration curves. The limit of quantification (LOQ; equivalent to sensitivity) was evaluated by injecting a series of increasingly diluted standard solutions until the signal-to-noise ratio was reduced to 10. The limit of detection (LOD) was estimated by injecting a series of increasingly diluted standard solutions until the signal-to-noise ratio was reduced to 3.

Table S4. LC–MS/MS conditions for quantitation of caffeoyl-, dicaffeoyl quinic acid derivatives (CQAs) by negative ion MRM mode.

**Compound**	**MRM transition**	**R^2**	**Regression line**	DP	CE	EP	CXP	LOD μg/mL	LOQ μg/mL
3-caffeoyl quinic acid (**2**)	353 -> 191	0.99	$y= 1.48e^3x -3.82e^4$	-60.0	-24.0	-4.0	-17.0	0.002	0.011
5-caffeoyl quinic acid (**6**)	353 -> 191	0.99	$y= 768x + 5.59e^5$	-60.0	-24.0	-4.0	-17.0	0.003	0.013
1,3-dicaffeoyl quinic acid (**9**)	515 -> 353	0.99	y=0.001 + 262	-61.0	-24.0	-4.0	-38.0	0.005	0.019
1,5-dicaffeoylquinic acid (**17**)	515 -> 353	0.99	$y= 1.87e^3x -2.35e^3$	-61.0	-24.0	-4.0	-38.0	0.004	0.017
